# Let's make up a story: design and evaluation of an improvisational storytelling robot to foster older adults' meaning and purpose in life

**DOI:** 10.3389/frdem.2025.1691140

**Published:** 2025-12-02

**Authors:** Long-Jing Hsu, Swapna Joshi, Erin Seliger, Kyrie Jig Amon, Waki Kamino, Natasha Randall, Weslie Khoo, David J. Crandall, Abhijeet Agnihotri, Katherine M. Tsui, Selma Šabanović

**Affiliations:** 1 Luddy School of Informatics, Computing, and Engineering, Indiana University Bloomington, Bloomington, IN, United States; 2 Humanities, Social Science, and Communication, Milwaukee School of Engineering, Milwaukee, WI, United States; 3 Toyota Research Institute, Cambridge, MA, United States

**Keywords:** meaning and purpose, storytelling robot, improvisation, older adults, engagement, Wizard-of-Oz, autonomous robot design

## Abstract

**Objective:**

Improvisational storytelling can encourage older adults to reflect on their meaning and purpose in life by creatively bringing up valuable patterns and themes from their memories and experiences. Robots for storytelling with older adults have so far aimed to boost their cognitive function and social interactions, rather than a pursuit of meaning and personal reflection.

**Methods:**

We used empirical analysis of expert storytelling facilitators to design a Wizard-of-Oz (WoZ) operated robot that engages older adults in improvisational storytelling to tap into their meaning and purpose in life.

**Results:**

Our analysis of stories created by 16 participants and their interactions with the robot suggests that older adults reflected on family, relationships, and activities meaningful to them and engaged with the robot as a companion during the activity.

**Discussion:**

We provide design implications for an autonomous storytelling robot and discuss the potential for information collected during the storytelling activity to further foster exploration of meaning and purpose among older adults.

## 1 Introduction

Improvisational storytelling techniques such as TimeSlips and Life Story Work (George and Houser, 2014; Truong et al., 2019) enhance the meaning and purpose of older adults' [age 60+ (Older persons, 2020)] lives (Scott and DeBrew, 2009) by linking them to their past experiences and offering a creative outlet (Ansello, 2020). These storytelling activities are often led by trained expert facilitators in assisted living and memory care facilities to offer older adults an imaginative and creative experience free from judgment and cognitive stress, promoting a shared understanding beyond literal language (Basting, 2013). In these activities, older adults use prompts or themes provided by the facilitator, and often their past experiences, as starting points or sources of inspiration for creating stories in the moment (Basting, 2013).

Robots with embodied forms, life-like qualities, and understanding of human social behaviors (Breazeal, 2004) that interact with humans (Alač et al., 2011; Breazeal, 2004) can engage people in social interactions (Alač et al., 2011; Oertel et al., 2020). Social robots have gained significant traction in applications with older adults due to their intuitive interaction mechanisms, which include voice command, social cues, and expressive capabilities. The tangible social presence these robots offer appears to resonate particularly well with older adults, allowing robots to provide with fun and entertaining activities. Moreover, the adoption of cutting-edge technology such as robotics can also instill a sense of empowerment within older adults, and serve to shift societal perceptions about this age group, highlighting their ability to adapt and engage with innovative solutions. Although robots have been developed to facilitate and engage individuals in storytelling activities (Ostrowski et al., 2021), the focus of such projects has primarily been on enhancing older adults' higher cognitive functioning, preventing cognitive decline, and promoting positive social interactions and communication among older adults (Van Patten et al., 2020; Robinson et al., 2014). Going beyond this existing work, we conceptualized a robot design to support improvisational storytelling that encourages older adults to reflect on the meaning and purpose of their lives, while providing them with an engaging and enjoyable experience.

This paper presents our adaptation of human-led improvisational storytelling strategies for a Wizard-of-Oz (WoZ) operated humanoid robot (LuxAI, 2022) facilitator to engage older adults in improvisational storytelling sessions. We evaluated 19 sessions with 16 older adults at a memory care facility and found that the activity with the robot was effective in transitioning older adults between memory sharing and story creation, and allowed participants to reflect on various elements of meaning and purpose in their lives, including family, relationships, activities, religious beliefs, and careers. The activity also provided our older adult participants with an engaging experience. We present insights into the design of an autonomous robot for reflecting on meaning and purpose, including recommendations on the storytelling structure, nuanced facilitation techniques, and strategies for addressing technical challenges. We further point to the potential for a future robot to personalize recommendations for activities, routines, and social connections based on data collected during the improvisational storytelling sessions, enhancing meaning and purpose in older adults' lives.

Our study contributes by (1) introducing the first WoZ design of an improvisational storytelling robot for older adults, with empirical research based on observations of expert facilitators, (2) describing the design of a robot-mediated activity that allows for ongoing engagement through various image cues and prompts to gather information about an older adult's meaning and purpose in life, and (3) supporting the growing efforts of Human-Robot Interaction (HRI) researchers and designers to create robots that foster older adults' sense of purpose and meaning in life.

## 2 Background

Meaning and purpose are widely acknowledged as key components of wellbeing (Ryan and Deci, 2001), and social robots have emerged as significant enhancers of older adults' well being and quality of life (Alves-Oliveira et al., 2015; Chen et al., 2020). These robots foster engaging, enjoyable, and soothing interactions (Góngora Alonso et al., 2019), which may potentially bolster mental health and emotional wellbeing (Scoglio et al., 2019). Recent research (Randall et al., 2023a) highlights how social robots augment emotional support and social connectivity among older adults (Pu et al., 2019), thereby improving their overall quality of life (Alves-Oliveira et al., 2015; Chen et al., 2020). Furthermore, robots have been shown to strengthen social bonds and promote self-reflection, both of which are essential for psychological health in later stages of life (Randall et al., 2022). Therapeutic sessions have utilized pet-like robots such as AIBO and Paro to engage older adults (Chang et al., 2013), facilitate social interactions between staff and residents (Šabanović and Chang, 2016; Pu et al., 2019), and support community activities (Joshi and Šabanović, 2019). Additionally, humanoid robots like the Nao have been employed to actively involve older adults in group activities (Tanner et al., 2023). Recent studies with humanoid QT robot show how engaging older adults in self-reflection exercise with robot supported their desire to make positive life changes (Randall et al., 2023b). Overall, robots are known to cater to the needs of solitary older adults by enhancing cognitive functions, which indirectly supports their overall wellbeing (Van Patten et al., 2020). Given the substantial benefits social robots offer in enhancing life quality for older adults, they also present significant potential for enriching wellbeing indicators such as a sense of purpose and overall mental health.

In this section, we review previous research on the importance of having meaning and purpose in the lives of older adults and the benefits provided by participation in meaningful activities such as storytelling. We also highlight the unique aspects of the TimeSlips model, an improvisational storytelling approach that has informed our study, and point to prior work on robot design for storytelling that provides valuable insights and helps identify opportunities for development.

### 2.1 Meaning and purpose for older adults

Meaning has been defined as a sense of coherence in one's life, while purpose refers to having a clear intention or set of objectives or a goal and working toward it (Reker et al., 1987).

People's sense of meaning and purpose in life can change throughout their lifespan (Reker et al., 1987). For example, people make choices about their goals based on their internal states and capabilities (Boker, 2013). However, aging can result in a decline in meaning and purpose; for instance, older adults may experience smaller social circles from the loss of loved ones, retirement from work, decreased physical abilities, and other age-related changes (Irving et al., 2017). These experiences can lead to a sense of emptiness, loss of identity, and a decrease in overall purpose (Pinquart, 2002). Furthermore, as older adults face limitations and changes in their physical and cognitive abilities, they may have difficulty pursuing the goals and activities that previously gave them a sense of meaning and purpose (Zahodne et al., 2014). Previous research has shown that older adults who have a strong sense of meaning and purpose tend to have better physical and mental health outcomes (Musich et al., 2018; Krause, 2009), are more physically active (Kim et al., 2020), and have longer lifespans (Krause, 2009).

Older adults often find meaning and purpose through self-reflection, particularly by revisiting their past experiences to rediscover meaning in their lives (Reker et al., 1987). This process can bring both a sense of fulfillment and a sense of despair (McLeod, 2013). Meaning and purpose can occur at a personal, interpersonal, or community level (Randall et al., 2022). On a personal level, individuals can find meaning and purpose through activities like hobbies (Randall et al., 2022), things they enjoy, religious beliefs, and activities that they feel they can contribute to (Hupkens et al. 2018). At the interpersonal level, good relationships with family and friends can foster self-care and improve physical and mental health, leading to an overall sense of happiness (Mineo, 2017). Finally, social connections at the community level, such as with institutions and community organizations, can provide a sense of purpose (Randall et al., 2022).

Reflecting on one's life for meaning and purpose can be facilitated through activities and therapies led by professionals. For instance, reminiscence therapy, led by an expert facilitator, helps participants recall and reflect on different life stages, such as childhood, adulthood, and old age and emphasizes engagement with family, friends, and romantic relationships (Melendez Moral et al., 2015). Similarly, programs like intergenerational writing and exchange promote appreciation of people's lives by allowing individuals to understand their own experiences as well as others' (Chippendale and Boltz, 2015). These programs help individuals to identify patterns, themes, and values that have been important to them over time, and along the way provide a sense of meaning and purpose. Additionally, by sharing and exchanging life stories, people can self-reflect and build connections and relationships, which can be a source of support and fulfillment in their lives (Kindell et al., 2014).

### 2.2 Meaning and purpose through storytelling

Storytelling has been found to enhance meaning and purpose in older adults' lives (Scott and DeBrew, 2009). Through storytelling, older adults can share personal experiences, recall past events, and engage in creative activities that increase their sense of social connection, elevate wellbeing, and give them a greater sense of purpose (Cappeliez and O'Rourke, 2006; Csikszentmihalyi, 1997). Storytelling also offers older adults opportunities for self-reflection, creative expression, and personal growth (Scott and DeBrew, 2009).

Storytelling is beneficial not only for older adults but also for caregivers, healthcare providers, and facilitators listening to their stories. Listening to personal narratives can provide a deeper understanding of older adults' emotions, memories, and experiences (Scott and DeBrew, 2009). For instance, studies have shown that storytelling has helped nursing and medical students gain a better understanding of older patients and become more comfortable talking with them (Scott and DeBrew, 2009; George et al., 2011). This leads to better care and support for older adults' pursuit of meaning and purpose in life.

However, memory decline is common in aging, so older adults may find it challenging to reminisce about personal stories (Park and Festini, 2017). To overcome this challenge, TimeSlips is a popular form of improvisational storytelling activity that encourages older adults to incorporate their experiences and memories (TimeSlips, 2022) in creating stories. It is conducted in group and one-on-one settings with certified facilitators using visual image cues and verbal prompts to encourage creative expression. The facilitators are trained in storytelling moderation techniques and have access to resources, practice and evaluation methods, and feedback from experts. The facilitators are trained to value and respond to older adults' remarks, and put effort into maintaining their enthusiasm (Loizeau et al., 2015), use specific prompts, and repeat back and affirm older adults' responses (TimeSlips, 2022). This results in positive feedback from older adults who find the facilitators to be pleasant, stimulating (Loizeau et al., 2015), and at times the only motivation to participate in storytelling activities (Loizeau et al., 2015). Studies show that facilitators can also increase older adults' engagement in social interactions with others in a group during the storytelling activity (Fritsch et al., 2009).

While the TimeSlips storytelling method involves a human facilitator, other storytelling methods involve exchanging written stories through postcards (Bennett et al., 2015), human-mediated phone sessions (Regenbrecht et al., 2022) and video chat (Regenbrecht et al., 2022). However, some of these methods pose challenges for older adults due to age-related language difficulties (Burke and Shafto, 2004) and decreased dexterity for writing (Goodpaster et al., 2006). Robots offer an alternative medium for storytelling by providing an interface that can be tailored to meet the needs of older adults, making the experience more accessible and enjoyable for them (Frennert and Östlund, 2014).

### 2.3 Robots for storytelling

Robots with embodied forms, life-like qualities, and potential for human-like social behaviors (Breazeal, 2004) offer possibilities for unique engagement and storytelling, setting them apart from other interactive systems. Robots have been used for storytelling in many scenarios, including children's entertainment, museums, language education, therapy, elderly care, persuasion, and entertainment (Ostrowski et al., 2021; Ham et al., 2015). They offer experiences through their expressions, movements, tone of voice, and other forms of interaction, with the purpose of educating, entertaining, improving language abilities, and providing therapy and care to various groups of people (Shibata and Wada, 2011).

One prior study on robots for storytelling suggests that people prefer a format of storytelling facilitation in which a robot reads the storytelling activity and another robot supports people as they answer the robot's questions (Tamura et al., 2017). (Ali et al. 2019) designed robots to encourage storytelling through a game, where the robots took turns with the participant using verbal and non-verbal cues such as wonder, curiosity, excitement, and surprise to prompt the participants to express their creativity.

A study by (Leite et al. 2017) found that users preferred robots that could retain information from previous conversations and use that information in later storytelling sessions (Leite et al., 2017). Another study by (Wu et al. 2019) used deep learning techniques to enable a robot to ask questions about personal photographs and understand the associated personal stories (Wu et al., 2019).

Prior studies on storytelling robots have primarily focused on children as the participants (Kory and Breazeal, 2014; Elgarf et al., 2021; Ali et al., 2019). Nevertheless, there have been some storytelling robots designed for older adults with a focus on cognitive training (Tokunaga et al., 2019), therapy, or for providing companionship to help combat feelings of isolation (Miller and McDaniel, 2021). One example (Tokunaga et al., 2019) involves a robotic conversation-based system to encourage older adults to record short stories to be played back to other older adults (Tokunaga et al., 2019). Another study incorporated story collection prompted by photographs and recording limited to a short period of time (Mihoko, 2018). A third study used a robot to narrate stories to older adults using photos provided by the participants or their loved ones (Abdollahi et al., 2017). However, in these studies the limited recording time or the use of generic photos as prompts might have resulted in less in-depth and personal stories, potentially affecting the participant's connection with the story and their own sense of purpose (Abdollahi et al., 2017).

In summary, previous research highlights the importance of meaning and purpose for the wellbeing of older adults and the benefits of storytelling, particularly the TimeSlips model, through improvisation, self-reflection, and stronger social connections. While robots have been used for storytelling activities such as recording stories or providing prompts, they have less frequently been used for improvisation or self-reflection. Our goal in this paper is to design a robot that uses the TimeSlips improvisational model to support the meaning and purpose of older adults, building on prior research that considered the impact of different formats of robot facilitation and retaining information from previous conversations to enhance the storytelling experience.

## 3 Robot interaction and activity design

We learned about the TimeSlips method of storytelling through our participating eldercare community which uses it regularly with residents. TimeSlips uses visual cues and verbal prompts to encourage creative storytelling around a specific topic. To promote meaning and purpose, we incorporated the TimeSlips protocol into our robot-facilitated activity design.

We began by observing four sessions of TimeSlips-based storytelling sessions in our participating eldercare community: two led by the director of program development and education facilitator (F1), and two improvisational storytelling sessions with a focus on promoting meaning led by a certified expert facilitator whom we invited (F2). The sessions were analyzed to gain insights into how the expert facilitators engaged and prompted the older adults to create and share personal narratives, which informed the design of our robot-led “Wizard-of-Oz” activity.

### 3.1 Observations from human facilitation

We observed that the improvisational storytelling sessions followed a somewhat similar protocol that helped us identify key elements to be incorporated into our robot-facilitated activity:

#### 3.1.1 Image-based cues

Both expert facilitators (F1 and F2) used images as anchor points for improvisation, sparking conversation with the older adults. The themes of the images varied and included scenes of children, pets, and current events and seasons (such as a Thanksgiving photo in November). While facilitator F2 mostly used the official Timeslips resource library (Creativity Center, 2023) of images and related prompts, our community facilitator F1 used pictures from newspapers and magazines that she thought would be of interest to older adults.

#### 3.1.2 Question prompts and non-verbal behaviors

After presenting the chosen photo to the older adults, both facilitators asked open-ended questions about it, such as “*What do you see/smell/hear in the picture? What is happening in the picture? Is there anything (anyone) we don't see? What are they thinking (saying)? What is going to happen next?”*

One facilitator (F2) asked the older adults to interpret the image creatively, for example by asking “*Where do you want to say this is taking place?”* In order to incorporate aspects of meaning and purpose related to the older adults' preferences and meaningful experiences, the expert facilitator prompted reminiscing about the older adults' own lives. For instance, the facilitator noticed the older adults talking about their families and encouraged them to share more details, such as “*What did you say? Something about being (a family member)?”*

Both expert facilitators employed strategies to keep the conversation flowing around the picture and the story. They asked follow-up questions relevant to the older adults' responses, such as “*Do you have any children in your family that were that age once?”* and then “*Who was it?”* The facilitators also used generic prompts, such as “*Are you wanting to add more details?”* and “*So tell me more about that*.” When older adults struggled to answer, the facilitators encouraged them to improvise and continue.

While facilitator F2 utilized the prompts from Timeslips resources and asked generic open-ended questions to encourage deeper conversation, the community facilitator F1 had a deeper understanding of the older adults and was familiar with their interests, family situations, and experiences and used this knowledge occasionally to enhance engagement.

#### 3.1.3 Storytelling format

The expert facilitators followed a semi-structured protocol for conducting the storytelling sessions. They started by building confidence by getting the older adults' attention and warmed up the session by introducing themselves, introducing others present in the room, giving out image cues, and reminding them of the purpose of the activity. They also read the resulting story from the previous storytelling session to demonstrate what was expected. One of the facilitators (F1) emphasized that older adults could use their own experiences and imagination without worrying about being perfect.

During the storytelling activity, the expert facilitators used active listening and prompting techniques adapted from TimeSlips. They introduced the picture by saying, “*Just look at the picture. Yeah, it's telling me what's happening.”* The facilitators listened closely to the older adults' responses and asked follow-up questions to keep them engaged.

To promote active engagement, the expert facilitators repeated and summarized the older adults' ongoing stories to ensure the participants were aware of their involvement in the storytelling process. This encouraged participants to delve deeper into their experiences and imagination to create more detailed and meaningful stories. The facilitators then summarized the ongoing story, incorporating the older adults' creative responses and memories, and sought feedback to continue to engage them.

At the end of the activity, the expert facilitators brought it to closure by asking the older adults to come up with a title for their story. This helped the participants gain ownership of their creation and reflect on its most meaningful, creative, and retrospective aspects. It also provided a sense of completion for the older adults and ended the session on a positive note. The expert facilitators expressed appreciation for the older adults' efforts by saying something like “*You have written a great story,”* and thanked the older adults for their time and effort.

#### 3.1.4 Expert facilitator behavior

Both facilitators meticulously recorded the older adults' stories by taking written notes, without any use of recording devices. To show engagement and interest (while wearing surgical masks post-COVID-19), they made eye contact with the older adults and nodded during the storytelling process between prompts. This helped to create a positive and supportive environment for the older adults to share their stories and experiences.

### 3.2 Implementing the improvisational storytelling robot

#### 3.2.1 Robot

**Figure 1:**
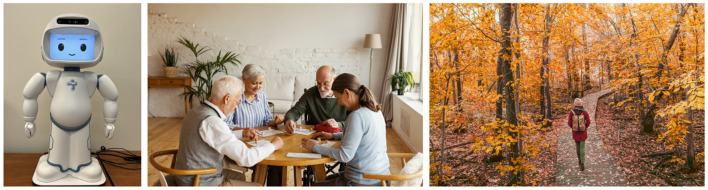
**(Left)** QT robot. **(Middle)** Group playing bingo. **(Right)** Person walking in a fall forest. Middle and right images were used as actual stimuli for the study. Maridav Bingo and Comeback Images - stock.adobe.com.

Our study used QTrobot (LuxAI, 2022) (resembling “cutie” and abbreviated as QT in this paper), an expressive social robot designed to support human-robot interaction research (Pinto Costa et al., 2019) (example of robot can be found in Figure 1). QT is 25 inches tall, weighs 11 pounds, and has a humanoid form with a screen-based face that displays facial expressions. It has expressive arms and a movable head. It works with a tablet that supports wizard-operated controls. QT has been previously used in various human-robot interaction studies, including music therapy, rehabilitation, and social connection (LuxAI, 2022) activities for older adults.

Our design of QT's interaction as an improvisational storytelling facilitator was informed by insights from our observation of the two above-mentioned expert facilitators regarding the format, strategies, image cues, and prompts. As a first step toward developing an autonomous robot, we created a Wizard of Oz (WoZ) interface to control QT's behaviors, as WoZ is commonly used in HRI studies as a step toward developing an autonomous robot (Martelaro, 2016). The WoZ interface allowed us to evaluate the need for adaptability in the interaction design (Kelley, 1984). This approach allowed us to gain a deeper understanding of the role of a human wizard in controlling the robot's interactions, and make necessary adjustments to ensure an easier transition to an autonomous system in the future.

#### 3.2.2 Preparing image cues

We also drew inspiration from the Timeslips resource library (TimeSlips, 2022), which offers photos of a variety of themes such as relationships, food, holidays, nature, and animals. In addition, for our selection of image cues, we considered literature on what provides meaning and purpose in life for older adults (Randall et al., 2022; Hupkens et al., 2018). We selected three image cues, based on inspiration from the Timeslips resource library and the potential to spark memories and encourage improvisation related to older adults' meaning and purpose through different levels of social connection (Randall et al., 2022; Hupkens et al., 2018): individual, family, and community. For instance, “a boy with a boat”—a picture of a child facing away from the viewer, holding a paper boat while sitting on a gravel shoreline by a lake—was chosen to evoke memories of cherished relationships from the older adults' childhood, children in their life, and activities in natural environments (examples of image cues can be found in Figure 1). The picture also provided an opportunity for imaginative storytelling by allowing the older adults to imagine things not shown in the picture, like the face of the boy, his intentions, and his family and social connections. The other two pictures depicted an individual and a community engaging in activities: “a girl walking in the forest” and “a group of elders playing bingo.”

#### 3.2.3 Robot application of prompts

Our design of the robot's behaviors and prompts aimed to foster older adults' engagement in the storytelling process (e.g., by asking “*What are they (people in the picture) doing?”*) and encourage them to share their experiences and thoughts. Other generic prompts were intended to enhance and deepen the older adults' engagement in improvisation (e.g., “*Tell me more about it.”*). Each type of prompt had 3–4 variations and corresponding pre-programmed robot behaviors (gestures and facial expressions).

#### 3.2.4 Adapting and modifying the protocol

We adopted the TimeSlips protocol used by expert facilitators to take into account the abilities and limitations of QT. For example, to introduce QT to the older adults, we added prompts for greetings and small talk, such as “*Hello there. I am QT, your robot friend. I am glad you are here.”* The robot then started the storytelling process by saying “*Let's make up a story.”* Since the robot could not summarize the story like the expert facilitator, it prompted the researcher in the room (wizard) to do so by saying “*I think we have made a nice story. Let's ask [wizard's name] to read it.”* The wizard then joins the conversation and summarizes the dialogue that the robot had with the older adult. QT showed appreciation and attention by saying “*Wonderful!”* often during the story and ending with “*Thank you for your feedback. This was fun for me.”* Finally, it concluded by saying “*Well then, have a happy rest of the day.”*

#### 3.2.5 Robot facilitation behaviors

We designed QT's social presence carefully in order to enhance the interaction with older adults despite QT's limited movements and size. We selected among the robot's pre-programmed facial expressions and gestures to make it more expressive and animated. The robot's mouth moved while talking, and its arms and head were also used for added communicative effect. At the start of the interaction, the robot smiled and blinked happily and kept its arms at its sides (see Figure 1). As it spoke, the robot's mouth moved to match its words. When greeting, the robot waved and said “*Hello there. I am QT, your robot friend,”* positioning itself as a friend to influence the older adult's behavior and encourage conversation (Kory Westlund et al., 2016). The robot moved its head to look at the picture with the older adults, raised its hand or placed its hands on its hips while asking questions, and at the end of the story, smiled and put its hands on its hips saying, “*I think we have made a nice story.”* To acknowledge the older adult's contribution, the robot clapped its hands and said, “*Thank you for your feedback.”*

#### 3.2.6 WoZ interface design

We used two iterations of the WoZ interface during the study. Initially, we used a web page-based interface that offered the wizard considerable freedom to choose from a wide variety of behaviors at any point during the session. The wizard could also input a custom prompt for the robot to perform. In anticipation of the robot's future autonomous capabilities, we later updated the WoZ interface using QT Studio's Blockly, a block-based coding interface (Developers, 2022). This iteration reduced some of the wizard's flexibility and limited their options, making it more suitable for translation to autonomous operation later on. This contrasts with more recent social robots that integrate LLMs to enable structured yet adaptive dialogue, a capability we plan to implement in future iterations. In particular, in this version, the wizard had the freedom to choose from prompts and behaviors only during the improvisation of the story and had no way to write custom prompts in response to unique interactions. In such cases, the wizard moved on to the next question or repeated the ongoing question without answering the participant's query. The study ends when the robot has completed its set of questions and says, “I think we have made a nice story,” after which the wizard joins the conversation, summarizes the dialogue, asks for feedback, and then says “Good-bye.” Despite the variations in the WoZ interfaces, the robot used the same prompts and was operated by the same wizard in all interactions.

## 4 Evaluation

### 4.1 Settings and participants

Our evaluation site was an assisted living and memory care facility with residents, daycare visitors, staff, and companion caregivers. The facility followed a person-centered care approach (American Geriatrics Society Expert Panel on Person-Centered Care and Brummel-Smith et al., 2016), accommodating individual needs through a personal selection of activities such as music, storytelling, prayers, exercise, intergenerational play, and research participation that was meaningful to the older adults. Followed by the human-centered approach and ensuring the older adults are comfortable (Hsu et al., 2023), the study was conducted in an open space, and other older adults could freely come in and out the space. This meant that sometimes other older adults were also in the same room, but they did not disrupt the study. They were there just to watch.

In our study, we partnered with a memory care facility near the university, and the facility director informed us that participants would be in the mild to moderate stages of dementia. Participants were selected based on their willingness to participate in the study and their cognitive level. As individuals in memory care for dementia receive support with daily activities (Hooyman and Kiyak, 2008), their levels of ability can vary. Participants with severe dementia were not included due to their limited ability to interact with the robot. The same robot was used across all sessions for participants with both mild and moderate dementia, and we aimed to ensure the robot was accessible for both groups. To ensure proper interaction with the participants, we collaborated closely with caregivers and nurses. While we obtained verbal consent and allowed participants to join and leave the study as they wished, we acknowledged the challenge of fully gauging their understanding of the activity and its purpose. Instead of directly asking participants to rephrase their understanding, which could feel interrogating, we took a different approach. First, we emphasized our role as students and researchers from Indiana University, clearly stating our intention to conduct research with them. Secondly, we confirmed their consent by asking if they were excited to participate in a robot storytelling activity and if we could videotape their interaction. Positive and enthusiastic responses were considered as consent.

### 4.2 Data collection

We conducted 19 storytelling sessions with 16 older adults [4 men and 12 women, ages ranging from 71 to 91, Mean (M) = 81.94, Standard Deviation (SD) = 7.75]. Three of the older adults voluntarily participated in a second session. The sessions usually lasted 15–30 min. The same wizard was in charge of all 19 sessions and had previously been involved in observations of expert-led storytelling sessions. All sessions occurred in public locations within the memory care facility. We acknowledge that the environment may have impacted the conversational flow, as older adults may sometimes not be able to hear the robot clearly. In such cases, the wizard would intervene and provide clarification when participants were unable to hear. The public space did not affect automatic speech recognition performance or errors (e.g., interruptions), as the wizard could decide what to say in the moment.

**Figure 2:**
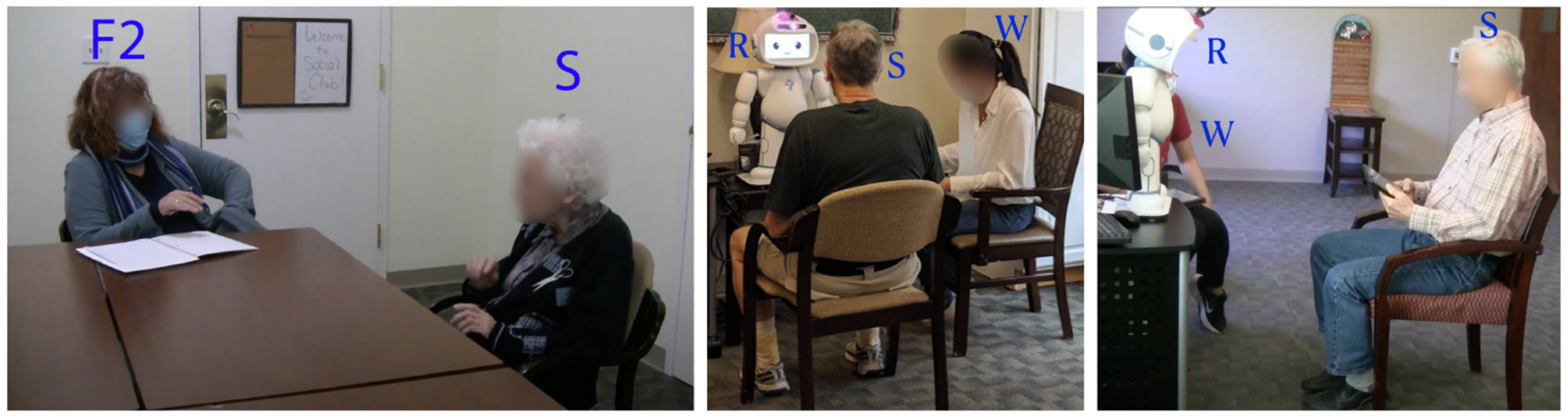
**(Right)** Storyteller older adult (S) storytelling with human facilitator (F2). **(Middle and Right)** Storyteller older adult (S) interacting with the robot (R) with a wizard (W) present.

Before each session, we welcomed the individual older adult into the study room at the facility, provided an overview of the session, and obtained their verbal consent. The older adult then started the storytelling activity with the robot. The wizard sat beside the robot and the older adult to manage the activity and offer additional support (Figure 2). Our decision to have the Wizard physically present in the same room, rather than operating invisibly, was driven by several considerations, primarily ethical and practical in nature. We chose not to use LLMs with contextual open dialogue because we wanted to control the words the robots used, as the use of these models may produce untrustworthy language or lack moral intent (Williams et al., 2024). We aimed to create an environment that closely mimics the real-world environment of the facility where older adults often receive assistance from caregivers or staff in technology interactions. This setup also allowed for immediate, visible support, which helped in making the participants feel more secure and comfortable, thereby facilitating interactions. For example, the wizard, repeated or rephrased the robot's questions to ensure they were understood, which mirrors the kind of assistance that is provided in the facility by staff members.

After the interaction, we solicited feedback from the older adults about the activity and the robot's performance. The questions concerned their experiences with the activity, reflecting on memories, especially on the significance of relationships with family and friends, and their desire for the robot to provide more opportunities for making up stories and triggering memories. Since the goal of this study was not to study health outcomes from the use of robots, we refrained from collecting any data on participants' individual health, such as their stage of dementia.

Lastly, we expressed our gratitude to the older adults for their participation. The interactions during the sessions were audio- and video-recorded both by the robot, as well as external camera for behavioral analysis. The study was approved by the institutional review board (IRB #12156) at our university.

### 4.3 Measures and analysis

Our evaluation aimed to assess the effectiveness of the robot in (1) facilitating storytelling sessions that promote reflection on meaning and purpose, and (2) providing an engaging activity. The main source of data for the analysis were transcriptions of the audio collected during interactions with the robot, transcribed manually.

To determine if improvisational storytelling with the robot prompted older adults to reflect on issues related to meaning and purpose, we transcribed the audio recordings of the storytelling sessions and performed thematic analysis by identifying and organizing the themes from our data (Braun and Clarke, 2012). Specifically, two authors initially generated codes through open coding of the data (inductive approach). During the coding process, three authors discussed the codes weekly and refined and reviewed emerging code categories and subcategories for similarities and differences with previous codes for 12 weeks. We did not measure inter-rater reliability, as we focused on identifying meanings and themes in the data, not on interpreting the qualitative data as having one “accurate” or fixed meaning (Braun and Clarke, 2021). As such, we identified patterns within the data and engaged in discussions among the three authors regarding our observations and coding decisions to achieve consensus. We continued these discussions until reaching saturation, focusing on reflections related to meaning, purpose, and engagement themes, as demonstrated in the findings and Table 1 The findings from the codes “Content of Story” reflect in subsection 5.2 (Findings/Reflections on aspects of meaning and purpose), and those from “Response to QT” and “Feedback” reflect in subsection 5.3 (Findings/ Engagement) below.

**Table 1:** Summary of thematic analysis codes and frequencies.

**Themes and codes**	**Instances**
**Reflections on aspects of meaning and purpose**	
Relating personal stories to elements in pictures	137
Imaginative and creative storytelling	195
**Engagement and reflection**	
Warm Hellos	16
Compliments	19
About the robot (age, family, origin, etc.)	8
Perform other tasks beyond the storytelling activity	24
Asking questions of the QT robot	119
Expressing similar opinions	72
Willingness to engage with the robot	173
Own limitation	57
Compliments and encouragements	201
Thank the robot	44
Gratitude toward robot	33
Farewell	28
Positive Feedback about experience	9
Impression of the robot's appearance and behaviors	4

## 5 Findings

On average, the participants interacted with the robot for about 16 minutes (SD = 6 min) per session. We begin by highlighting two examples of the robot's improvisational storytelling session. We then present findings from content analysis on how older adults shared memories and made up stories with the robot. Finally, we present observations from the older adults' behaviors and their interactions with the robot and their feedback on the activity.

### 5.1 Sample interactions

Our robot followed a structured protocol at the beginning (during greetings and familiarization) and the end of each session, but the sessions varied since the wizard selected prompts according to the ongoing flow of each participant's own story. Specifically, there was a sequence to follow in which the wizard could choose when to ask the next question and select simple responses such as “*tell me more about it*,” offering a compliment, or asking “*how was it like*?” Details of the conversation flow are provided in the Appendix. Below, we present exemplars from two participants, P1 and P14, to demonstrate the personalized approach of the robot in promoting meaning and purpose.

- Older adult P1 was given the “a boy with a boat” picture by the WoZ researcher. The robot began by prompting P1 to make up the story about the experience of the boy in the picture and then asked if P1 has similar experiences to prompt P1 to talk about their own life. To facilitate deeper engagement and personal reflection, the robot asked open-ended questions such as “*what is happening here?”* P1 described the picture as “*A real cute little boy is stooping down... a lake or pond. And he's got some paper boats I think he's probably made and he's putting them in the water.”* Recognizing the participant's fondness for children, the wizard had the robot follow up with questions to encourage further description, such as “*tell me more about it.”* P1 shared stories and mentioned that “*he's (the boy) wearing shorts and his mother likes him right near the lake,”* suggesting to the robot that the participant values the relationship between a child and a mother. The wizard then selected a prompt to evoke made-up content related to meaningful personal information about family, such as “*What are the family (mother) doing? Why are they there?”*. Toward the end, the wizard asked more questions to help P1 reflect on their own memories and experiences, such as “*have you been to Lake Lemon with your family?”* P1 replied by sharing more details about her family that were meaningful for her.
- P14 participated in a storytelling session with the photo of “a girl walking in the forest.” Throughout the session, P14 seemed to be drawing connections to her own life. She described the picture as “*It looks like a young woman is taking a walk through the woods on a beautiful fall day.”* The WoZ robot encouraged P14 to make up a story based on the image. In response, P14 said, “*There's only one person and she's walking with a backpack... This young woman will continue her walk until she goes off of the trail and back to whatever she's going to do next.”* To further prompt meaningful memories, the wizard asked P14 how relatable she found the picture. P14 was able to connect elements from her own life and shared, “*Yes, I've lived in wooded areas and in cities that had nice trails that went through wooded areas and I like to walk.”* However, the wizard noticed that at times P14 was uncomfortable with her responses and would frequently say “*I don't know.”* To make P14 feel more comfortable, the wizard made the robot compliment her responses, such as “*I loved it,”* and continued with more prompts which P14 willingly answered.

These two examples demonstrate the way in which the wizard tailored the storytelling experience for each individual and selectively chose prompts that helped shape the story, tried to ensure the older adult was engaged and comfortable in the activity, and encouraged them to reflect on meaningful aspects of their life while blending creativity with memories and experiences.

### 5.2 Reflections on aspects of meaning and purpose

As shown in the exemplars, older adults reflected on aspects of meaning and purpose in life with the robot. From a more detailed content analysis, we discovered that the older adults shared meaningful memories with the robot, made up stories, and synthesized their stories with specific aspects of their meaning and purpose in life.

#### 5.2.1 Explicitly sharing memories with the robots

As older adults reminisced about meaningful personal memories with the robot (*n* = 16, instances = 137), they shared about family structure, personal relationships, experiences and opinions of their own situation related to the picture, locations of their life, and memories of time spent with their family.

Six participants discussed the structure of their family including details such as the names and ages of their children and grandchildren. For instance, when prompted by a picture of “a boy with a boat,” P1 shared about her family: “*I'm 91 years old so my family is at this point is mainly four sons who were born in the middle of the, well, they were born in 1954, 56, 58, and 61...”* P1 then went on to talk more about the rest of her family members, saying “*I'm sure they'll bring a little [grandchild name]'s, the little grandchild, he was the closest to me, that bring him to see me probably my birthday in January.”*

Additionally, ten participants shared their experiences and feelings about their relationships with their family members. This often occurred when the robot asked for more information, and the participants were eager to talk about their families. For example, when shown a picture of “a boy with a boat,” P7 discussed her relationship with her family. The robot asked, “*how's your relationship with them,”* to which P7 replied, “*Oh, we all love each other... we have a good time. We work together and we play together. And we have lunch together. And sometimes we'd go on picnics together and go swimming or play ball. We have a very very good time. I love playing with my family.”* Similarly, P4 shared her close relationship with her family while telling a story about the “a boy with a boat” photo: “*I have very close relationship with my family. I have really good parents. And I have really good sisters and brothers.”*

The participants also shared their experiences and provided their opinions based on the pictures and conversations with the robot. For example, when interacting with the robot about a picture of “a girl walking in the forest,” P2 shared a similar experience: “*Oh well I've had similar experiences to this, but I've never been alone when I've done it... I think I would feel a little bit lonely if I were there by myself at her age.”* This provided us with the information that P2 preferred and had experiences walking in the forest with other people.

At times, the participants also mentioned experiences not directly related to the picture. For example, when shown the photo of “a boy with a boat,” P8 mentioned her religious beliefs. She stated, “*Man. You know Jesus Christ, our Savior created Earth.”* P8 went on to share her experiences at church, saying, “*I go to church when I can... you go to church, and you sit down in a pew which is place to sit and you sing songs about Jesus. And then the preacher comes out and starts reading stuff from the Bible.”*

The older adults also shared detailed memories with the robot. For example, when looking at a picture of “a boy with a boat,” P6 began warning the child to stay away from the water, “*there should be an adult at the swimming place. No matter what. That should come first for someone to look at a child, either the parent, an aunt or an uncle, or grandparent.”* When asked “*do you have any memories of that?”* P6 began sharing a personal story: “*When I was four years old, my parents took our pony to the lake... I... didn't know how to swim... and he (the Pony) just kept going deeper... And a man came along... he rescued me and I remember coming out of the water...”* She continued talking without further prompting. As another example, P11 mentioned his career while talking about the photo of “a group of elders playing bingo.” He said he “*was a professor in the University.”* When prompted with the question “*is community important to you?”*, P11 replied, “*Yeah, I am not a professor anymore because I'm too old to be a professor now. But it was, it was a job I liked. And I think my students found that they could understand things that they hadn't understood before.”* This shows the significance of his career on his life and how he thought it impacted others.

Participants also reminisced about locations and activities that were important to their lives. When conversing about the 'a boy with a boat' and 'a girl walking in the forest' photos, several mentioned specific locations that the pictures reminded them of. For example, when looking at the ‘a boy with a boat' photo, P1 spoke of a lake located in a specific state. Similarly, P2 talked with the robot about the ‘a girl walking in the forest' picture and reminisced about her past experiences taking walks in a nearby state park, stating “*I feel real good looking at this scene. Reminds me of the days when I used to take walks in the [Park name] in [County name] is the one I remember. We used to walk in a lot. That's not too far from here.”*

From the participants' stories about their family structure, relationships, personal experiences, opinions, detailed memories, and important locations, it was evident that they were reflecting on memories that held significant meaning and purpose for them.

#### 5.2.2 Using memories to imagine with the robots

Participants often seemed to use their own memories and experiences as a starting point or source of inspiration for imagining details (*n* = 16, instances = 195) and ages of character in the picture, relationships, locations, things of pride and value, and to devise a title for their story.

In one instance P1, who talked about the story of “a girl walking in the forest,” imagined the character to be of the age of her younger self: “*I think it's a woman and ...she might be about my age when I was in the 40s.”* She then made up stories after connecting the woman to her younger self.

Although the faces of the characters in the photos were not visible, participants imagined them to be feeling positive emotions such as invigorated, fresh, healthy, happy, satisfied, and content. For example, when looking at the “a girl walking in the forest” picture, P12 commented, “*people enjoying themselves it looks like, they look happy, they're excited about what's gonna happen.”* P9, while looking at the “a boy with a boat” photo, shared that “*he looks very happy, he's very busy.”*

When the robot asked what the participants thought the character in the story was proud of, seven of them imagined it to be the character's relationships and creativity. For example, when looking at the “a boy with a boat” photo, participants shared that the child was “*proud of his mom and dad (P8)”* and “*he's very proud of this boat...I'm gonna say his paper boats because I, I know his father, he probably helped to make the boat.”* Three of the participants envisioned a bright future for the characters in the images and suggested things of value and pride for them. For example, P7, when discussing the “a boy with a boat” picture, said, “*Oh, I think he has a very good future. He will go to school... study hard..., he'll be whatever he wants to be. Because he studied. But it looks like he's really got intentions do something magnificent,”* and continued, “*He shouldn't try and stay in one position. He should keep an open mind and see what the future lies for him.”* This showed that P7 valued education, hard work, staying open to opportunities, and continuous growth.

Seven participants imagined the characters in the picture to have family, especially in the ‘a boy with a boat' photo, even though no family members were in the picture. For example, P7 imagined “*They're not right here but they're there. They're in their house. Maybe sitting out in lawn chairs and watching him play. Or maybe they're having a wiener roast.”* Nevertheless, through such imagination, they expressed their belief in family and relationships; for example, P1 said “*his mother likes him right near the lake so it must be warm, and she's looking out the window to see where her young son is. And if she can't see him, I'm sure she'll come back for him to answer her, so she will know where he is.”* This has shown us how she believes a mother should take care of and be attentive to the young.

Often the participants related the relationships of the character to themselves. P2 had earlier mentioned to the robot that she liked to go for walks with her friends and later imagined the lone character in the “a girl walking in the forest” photo doing the same: “*She was she's probably thinking about her friends. And maybe she's going to meet some of them later for a picnic or something like that.”* P3, for the same image, suggested, “She (girl in the picture) is married to a husband. She and her husband are living by just the two of them. They have no children with them right now, they're maybe going to adopt some people.” We later learned that he had an adopted child in their family.

When prompted to talk about the locations in the photos, four participants related them to places close to the participants' own homes. Looking at the “a girl walking in the forest” photo, P11 shared “*She has a family. She lives in Vermont. In New England. She's going home. She's on the path.”* Others related it to the participating assisted living center they were currently at. For example, when P16 saw the “a group of elders playing bingo” photo, they said, “*This is in a room of the of social studies of where we are now.”* Yet others related to places they had been in the past; for example, P12 said, “*In Monroe County there are parks all over the area where you can hike, follow the path, and it's just very pretty out there.”*

Finally, when the robot prompted the older adults to reflect on and synthesize their responses by giving a title to their story, 11 participants captured some of the key themes they discussed and synthesized meaningful memories and/or imagination into the title. Those who shared more of their memories created titles related to themselves and their memories. For example, P2 mostly shared memories of her childhood with her parents, and came up with the title “*The youth of [her own name].”*. Similarly, P15 made a story about his wife and their kids and titled it “*[wife name] and [his name],”* while P16, who discussed his past career as a professor, named his story as “*How students and professors can mix.”*

### 5.3 Engagement

Most older adults were highly engaged in the process. We observed instances of positive engagement in the interactions between the older adults and the robot, reflections of the older adults, and some behavioral indicators.

#### 5.3.1 Interactions between the older adults and the robot

Although older adults were informed about the role of the Wizard as part of the research team, our observations indicated that they primarily focused on the robot during the sessions, treating the Wizard's interventions as similar to those of a facilitator rather than as part of the robot's functionality. This distinction was supported by our analysis of the video recordings, where it was evident that the participants' attention returned to the robot immediately following any intervention by the Wizard.

We observed during the activity that the QT robot acted as a facilitator that kept older adults engaged and interactive by promoting their memories and experiences. In response to the robot's greeting, older adults reciprocated with warm hellos (*n* = 16, instances = 16) and compliments (*n* = 15, instances = 19) such as “*Hi! Good to see you today, You're a cute little person”* (P4) and “*Hi how are you doing? Oh, big smile, Oh”* (P3). These responses showed the participants' pleasure in interacting with the robot.

As a form of engagement, the older adults showed curiosity about the robot and its capabilities. They asked questions such as where it came from, its age, and its name (*n* = 2, instances = 8). Additionally, they admired the robot's abilities and expressed interest in knowing if it could perform other tasks beyond the storytelling activity (*n* = 10, instances = 24). These included requests for the robot to sing a song, do math, play, teach, read the bible, go for walks, entertain people, and even wave back.

Throughout the storytelling sessions, the older adults demonstrated active engagement by frequently asking questions of the QT robot (*n* = 16, instances = 119). In response to the robot's prompts, they asked about the picture they were shown, and at times, used the pronoun “we” to suggest a companion-like relationship with the robot, asked for the robot's assurance, and wanted to know how the robot felt about something in the picture. They acknowledged the robot's prompts by repeating them or expressing similar opinions (*n* = 13, instances = 72). They also reciprocated the robot's prompts by asking the robot questions back, demonstrating their belief that the robot was a participant in the activity. For example, when the robot asked if a character in the picture had a family, P12 asked back “*Do you know whether they have a family?”* These instances of active engagement suggested how older adults viewed the QT robot as a companion in the storytelling experience.

At times, the older adults encountered difficulty in understanding or hearing the robot, they would either repeat what they thought they heard or request clarification, such as “*Say again”* (P15). Older adults also politely acknowledged distractions, such as “*Pardon me? I was distracted by someone who came to the door. What's your question?”* (P1), and expressed eagerness to continue working with the robot. Despite the hearing difficulties, older adults demonstrated a willingness to engage with the robot during the storytelling activity (*n* = 16, instances = 173).

We found that 15 out of 19 sessions involved participants responding directly to the robot's prompts during the storytelling activities. However, there were instances in which they found the task challenging and were unable to come up with an answer. In such cases, they often conveyed that it was due to their own limitations and not the robot's failure as a facilitator (*n* = 10, instances = 57). For example, P10 said “*I'm not very good at making up things”* and P12 said “*Now I'm not very good at this picture business.”* When asked to imagine and come up with something creative, some felt overwhelmed and could not answer, and implied that the difficulty was due to their cognitive limitations. For example, P4 said “*I can't think of anything at the moment. I might later... My head's a little foggy today.”*

The older adults frequently responded positively to the robot's compliments and encouragements (*n* = 16, instances = 201). They thanked the robot (*n* = 16, instances = 44) and acknowledged its support in their contribution, such as “*Thank you for coming and making it possible”* (P1) and “*Nice work on your part”* (P5). They especially expressed joy when the robot deemed their response as “wonderful,” with comments like “*Oh, you love it... Oh, I love you. You are wonderful,”* (P13) and “*Wonderful, oh I'm so happy to hear that”* (P8).

At the end of each session, the older adults frequently expressed their gratitude toward the robot (*n* = 13, instances = 33) and bid farewell (*n* = 13, instances = 28). For instance, P13 thanked the robot, saying “*Thank you for having a conversation with me. I appreciate that.”* Similarly, P1 also showed appreciation for the activity, stating “*I think this has been a lot of fun. I enjoy this project that we're involved in and thank you.”* These instances demonstrate the participants' recognition of the robot's interactions and their perception of the robot as a companion.

#### 5.3.2 Older adults' reflections on the activity

We collected feedback from the older adults on the activity and found that overall, most (n=9) were engaged in the activity and had positive feedback about their experience. For example, P14 said “*I think it's been fun. I didn't know what to expect but it's been nice for me to think back on how I enjoyed walking through beautiful green spaces.”* She described how it also reminded her to get out with some friends: “*Well, one thing that really hit me is I need to get out of [care facility] with friends and go walking in the woods.”* In another example, P2 said “*I think it was very pleasant... thing to do. Very nice... because it's not in any way worrisome. You know, it's a good way to relax.”* Similar sentiments were also aired by P1, P6, P10, and P14. Two older adults seemed indifferent about the activity and were not very excited about the thought of doing it in the future.

The older adults also shared their impressions of the robot based on this activity. Two older adults (P6 and P9) talked about the robot's appearance and behaviors, suggesting they liked its eyes and smile. Two others gave remarks on its behaviors and movements. P14 mentioned talking to the robot was like talking to a “*very intelligent child”* and thought the robot was good-hearted, friendly, and had thoughts of its own. P11 called it an “animated figure.” P13 commented on its facilitation of the activity, “*Oh, I love this. I love the robot and I wasn't expecting the spontaneous remarks from the robot.”* None of the older adults mentioned anything about the robot being wizarded or controlled by the researcher in the room. Nevertheless, the positive feedback showed that the older adults generally enjoyed the interaction and were engaged in the process.

## 6 Discussion

Improvisational storytelling can connect older adults to their past experiences and allow them to reflect on relationships and activities that bring purpose and meaning to their lives (Scott and DeBrew, 2009). In addition, storytelling is an inherently beneficial activity for older adults and allows people around them to gain insight into their connections and experiences. Our aim was to support older adults' meaning and purpose through engaging robotic prompts and reflections. We used the Timeslips model with a Wizard-of-Oz robot, incorporated literature on meaning and purpose into the design, and adapted image cues, prompts, and protocols used by expert facilitators into robot behaviors in order to enhance its sociality. Since our long-term goal is to develop an autonomous robot, we gave the wizard limited options in operating the robot so that we could more easily automate these behaviors in the future. We did not expect the same results as a human expert facilitator but instead viewed this work as a demonstration of the potential for promoting meaning and purpose through improvisational storytelling facilitated by a robot.

Our study supports previous research on creativity and memory sharing based on TimeSlips (TimeSlips, 2022; Gaughan et al., 2016). Our work contrasts with previous studies that have used robots in reminiscence or storytelling contexts. For example, Pepper used in a data-driven conversational interface with healthy older adults (Céspedes et al., 2022) was tested with participants who were not living with dementia, which differs from the current study. Other studies primarily focus on the design of the robot as the conversation host (Tucci et al., 2024) without providing detailed analyses of the conversations themselves. Furthermore, prior work often focuses on recalling a single memory rather than encouraging imaginative, multi-memory conversations, whereas the present study examines conversational detail, thereby complementing and extending prior research.

Our results showed that older adult participants found the activity engaging and that they produced creative responses to the robot's prompts. They incorporated their memories and experiences related to meaning and purpose, particularly on topics such as relationships and activities (such as visiting places, playing with friends, and going for walks).

### 6.1 Insights from the human-robot interactions

Similar to prior literature on human-facilitated Timeslips studies, our participants admired their facilitator (the robot) and were able to create, reflect on, and synthesize their stories with the robot's prompting, except in some instances where the participant found the task challenging and was unable to come up with an answer.

However, we observed that the experience of robot improvisational storytelling was fundamentally different from human-facilitated storytelling. During expert human facilitation, the environment seemed formal and the older adults were focused on providing their best response to a given prompt, while during robot facilitation, they were more casually engaged as they created the stories. Our observational analysis showed that the robot seemed to engage and capture the attention of the older adults throughout the activity.

Several older adults explicitly mentioned that they thought the robot was a “cute” and “childlike” but also an “intelligent” companion and collaborator. With the character set in mind, the older adult engaged with the robot in a natural but informal way. Their informality with the robot was also obvious when they greeted the robot, asked it questions, apologized to it for not being able to respond, seemed eager to hear its opinions, and thanked it for its work. Despite any technical issues, the older adults continued to engage with the robot to have a successful storytelling experience. Our findings support prior research that robots can support conversations and prompt their users for creative tasks and robot-facilitated storytelling can be engaging for older adults (Leite et al., 2017; Tamura et al., 2017).

The older adults' engagement with the robot seemed to facilitate sharing and creating meaning and purpose through storytelling. Similar to the ways in which they shared memories, they were willing to express their imagination related to the photo prompts, including the behaviors, feelings, and emotions of the depicted people including their demographics, character, names, and relationships. While making up stories with the robot, participants seemed very positive about the characters' feelings and emotions, their imagined futures, and their values such as hard work and exploration for achieving success in life. Additionally, they came up with names for the characters and invented relationships with others, often imagining the characters as having family nearby. Throughout the activity, most participants openly shared personal information such as their hopes, desires, and feelings about different members of their family. The older adults in our study effortlessly switched between conversations about memories and imagination, linked by meaning and purpose, during the storytelling activity with the robot. The connection between the older adults' memories and imaginative responses was evident, with memories serving as a source of inspiration and shaping the creative process (Schacter et al., 2012). This resembled a conversation-based therapy session (Erber, 1994) in which conversations flowed naturally as if older adults were speaking with another person. The robot encouraged them to reminisce, revisit positive memories, share meaningful moments in their lives, and talk about things of value to them.

The concept of talking about past memories connects with past studies of storytelling methods (Thomas and Killick, 2007; Scott and DeBrew, 2009) and reminiscence therapy (Melendez Moral et al., 2015) that elicit meaningful elements in people's lives. In contrast to these previous studies that were based on story-telling with human facilitators, our study used an engaging improvisational storytelling robot. This improvisational storytelling with the robot brings opportunities for further activities with the robot to foster more meaning and purpose in older adults' lives. As meaning and purpose for adults change and sometimes decline as they age (Irving et al., 2017), our findings showed how robots and storytelling could promote older adults' reflection of meaningful memories and create creative content.

### 6.2 Implications for the design of autonomous robots

Although our study used a Wizard-of-Oz design, our longer-term goal is to develop an autonomous robot that could automatically guide older adults through the improvisational storytelling process. Older adults' engagement and openness with the robot likely depends on multiple factors including the robot's appearance and form, the story-telling model, the image cues, and the wizarding. In an ideal situation, the envisioned future iterations would not rely on the WoZ setup. Instead, the system would operate autonomously in real-world, unsupervised scenarios that require minimal assistance from care partners. Their role would primarily involve supervising the process to prevent unprecedented outcomes, such as eliciting bittersweet emotions (Hsu et al., 2025). Our findings pointed to several opportunities for improvement, as well as valuable takeaways for the future development of an autonomous version.

#### 6.2.1 Activity structure

Our robot's interactions with the older adults used a structured format—starting with greetings, building up the characters and the story, and ending with a title to the story—inspired by a human-led improvisational storytelling activity. The structure made it easier for both the older adult participants to understand the activity's flow, and for the wizard to choose appropriate prompts suitable to the ongoing flow of the activity (Lee et al., 2010). This structure also allowed the wizard to systematically document the ongoing story, in order to summarize it at the end of the activity to promote the participants to reflect on meaning and purpose.

Furthermore, our shorter duration of 16 min demonstrates a more concise interaction time. In the HRI community, interactions between conversational agents and participants are typically brief. Previous research has investigated interaction lengths of 20 min (Yamazaki et al., 2019; Feng et al., 2022) to 30 min (Cruz-Sandoval et al., 2020), to prevent fatigue from participants living with dementia and avoid disruptions from staff or visitors.

We expect this structure will make it easier to develop an autonomous robot to lead the interaction, as it can follow pre-coded activity structures.

#### 6.2.2 Nuanced facilitation

The seamless switch between conversations about memories and imagination could be attributed to nuanced facilitation by the robot. Our wizard often alternated the robot's prompts between memories and imagination in a way that complemented the conversation flow. The switching also helped to encourage greater engagement on particular topics, to reveal the topics' significance for the older adult's meaning and purpose, and to balance creativity and sharing of personal stories. An autonomous robot would require an understanding of when to make such a switch. This could be done by asking for appropriate prompts when catching the specific details of the words related to their memories (i.e., my grandson, my dog, I remember) and imaginations (i.e., that girl, that person in the picture). One possibility is to use LLM-based prompt engineering to manage conversation flow with pre-scripted questions, followed by LLM-generated responses (Hsu et al., 2024) that encourage creativity and the sharing of personal stories. As such, this approach could help prevent the risk of the robot (LLM) going too deeply into sensitive topics, and having a care partner there to safeguard could be helpful.

#### 6.2.3 Use of image cues

Our use of specific image cues for the activity seemed to promote the older adults' reflection on specific aspects of meaning and purpose, such as family, relationships, and activities that were key elements in a person's life (walking, socializing with others). Older adults in our study shared a variety of personal memories with the robot during the sessions, such as their own family structure, personal relationships, experiences, and places that were important to them. However, most responses were about family, relationships, and people, suggesting that family and relationships are of greater value to them than activities or places. This finding enhances the potential of storytelling with specific image cues (TimeSlips, 2022; Wu et al., 2019) to add another dimension of meaning and purpose in their lives. We also found that a few times our use of image cues led to discussions about meaningful aspects of older adults' lives that were not directly related to the images, such as their religious beliefs, career experiences, and significant events. This concept can be applied to an autonomous robot by providing it with a personalized bank of prompts for each image, based on its prior knowledge about the person, including their meaning and purpose in life. The robot could also request images from the user to capture a broader range of meaning and purpose, covering topics such as relationships, activities, hobbies, and community service (Randall et al., 2022). An expert facilitator could input well-crafted prompts into the robot, and the robot could use facial expressions and gestures to go along with these prompts.

#### 6.2.4 Summarizing the story

The wizard's summarization at the end of the session provided the participants with a chance to feel a sense of accomplishment and to reflect on their meaning and purpose in life. This practice was adopted from TimeSlips storytelling, where facilitators write down notes during the storytelling session and later read the narrative to the participant (George et al., 2011). Although in our study, this component of the story-telling protocol required the presence of a wizard, an autonomous robot could incorporate Large Language Models (LLMs) to summarize the storytelling and retell the participant's story while emphasizing the significance of meaning and purpose in the person's life. This story could be created by gathering keywords from the story's plots and elements related to meaning and purpose, and could also incorporate prior knowledge of the person's life and image cues.

#### 6.2.5 Robot gestures and emotions

The older adults' feedback revealed that they noticed and appreciated the robot's behaviors and gestures, including its smile, eye movements, and small talk conversations, and that these behaviors led them to think of the robot as a companion or friend. Their feedback indicates that a robot's social presence, such as its ability to talk, gesture, and make eye movements, can enhance users' enjoyment of the storytelling activity (Heerink et al., 2009). This finding supports previous research that shows that exaggerated movements and expressions can increase social presence and improve user engagement (Lee et al., 2006). The autonomous storytelling robot should thus incorporate exaggerated gestures and facial expressions, while also aligning them with the flow of the activity or the questions being asked. For example, the robot could point to the photo when talking about it, or clap its hands and show a proud face to indicate appreciation when the older adults create a title for the story.

#### 6.2.6 Recognizing human emotions and responding appropriately

The human wizard watched the participant's behavior and caused the robot to respond accordingly, which improved the flow and engagement of the conversation. For example, older adults sometimes encountered difficulties in comprehending the robot's prompts and seemed overwhelmed or confused. In such situations, the wizard would intervene to repeat the prompts as needed or select prompts for the robot to move to the next question. An autonomous robot should be able to recognize the emotional state of users by recognizing spoken and nonverbal speech cues, analyzing emphasis, gauging response length, and observing facial expressions (for confusion) and adjust its interactions accordingly. The autonomous robot could also ask follow-up questions to check whether the older adults are understanding what was communicated by the robot.

For example, if an older adult shares something about a friend or family member who passed away or expresses worry about something in a photo, the autonomous robot should respond with empathy and use appropriate prompts to console older adults, pause the activity, and provide a listening ear before eventually trying to connect back to the positive memories and stories or point toward other meaningful aspects of life (Iglewicz et al., 2020).

#### 6.2.7 Response to unanticipated questions

Our use of a wizard and a pre-set list of prompts limited the robot's ability to respond to unique questions posed by older adults. For instance, the robot was unable to answer questions about its own capabilities. This inability to answer unanticipated questions could have interrupted older adults' engagement in the activity. This could be resolved by incorporating a conversational AI model to allow more free-flowing conversations. This would help the older adults understand more about their robot facilitator and could build a stronger relationship. The robot should also set expectations and provide more information about itself, including its capabilities and limitations. These kinds of expectations could also carry over to future conversations and may inform changes in robot design to prevent the novelty effect.

#### 6.2.8 Consent from the user

Our study collected data from older adult participants for research purposes, with their consent obtained before participation. We reassured the participants that the data was shared with the research team for analysis and synthesis. However, using an autonomous robot without human oversight raises ethical concerns that need to be addressed in future research. Privacy and data collection are key concerns, as users or their families and caregivers may be unclear about the intent for data collection, and how their data will be processed by the robot. To address this, future autonomous robots, especially those that are commercially available, may need to regularly remind users about their privacy and data collection, such as at the start or during the activity when the robot summarizes the story (Fosch-Villaronga et al., 2020).

### 6.3 Potential for future research

Aside from the future development of the autonomous robot for improvisational storytelling, our study has laid the ground for future research by (1) addressing limitations for similar research, (2) pointing to potential use of data for meaning and purpose, and (3) creating opportunities for applications to other use contexts.

#### 6.3.1 Addressing limitations for similar future research

Our research was not without limitations. The use of novel technology always raises the possibility of unintended consequences, and our application is no exception. Unintended consequences could include reduced human-to-human interaction, feelings of loss of control, and concerns of sharing too much information, leading to loss of privacy (Sharkey and Sharkey, 2012). Technical issues (connection to the internet, bugs in programming, imperfect AI models, etc.) (Toscano, 2011) could lead to a potential lack of trust in robots. While we aimed to address these issues in our conceptualization, such as by suggesting how the goal of our research is to provide additional activity with robots for older adults and by describing the design of activity as individualized to ensure data privacy, there is much research going on in these directions, on trust in HRI and ethical concerns that can bolster future development of an autonomous robot. Future research could explore these limitations, address them proactively, and enhance the technology to mitigate any negative effects in the context of a memory care facility.

Furthermore, our research did not collect logs, systematically log frequencies, health data, and record instances in which participants struggled to engage verbally or to formulate responses. We also did not conduct further analysis of speech and multimodal behavior data from the recordings to relate them quantitatively to engagement (i.e., gaze, posture, or facial expressions); however, this presents an avenue for future work to more deeply explore how robots can further engage with and support people living with different levels of dementia. For example, we suggest that future research could enable the robot to log its own interaction instances, as well as multimodal behavioral data from other connected devices. This would allow future autonomous systems to more systematically calculate and analyze the effects of technology.

Our study also did not address the novelty effect, which may occur when older adults first encounter the robot or have seen it before but do not remember it. The novelty effect is common, especially in relation to the robot's embodiment and social presence, as it provides participants with a new experience and people might interact differently over a longer period of time (Smedegaard, 2022). Nevertheless, our short-term interaction was meant to provide initial results on the feasibility and usefulness of our interaction design, and we considered its short duration may help prevent older adults from becoming overly adapted to the robot (Andriella et al., 2020) while also providing immediate results. Future research should aim to better understand this phenomenon through a longitudinal follow-up study.

One participant described the robot as “an intelligent child.” This observation could raise concerns about infantilization, a form of abuse in which older adults are treated like children (Noriega et al., 2023). Although this was neither intended nor specifically observed in our study, future research should carefully consider making sure we or the robot do not speak to the older adults living with dementia as if they were a child.

#### 6.3.2 Use of data for meaning and purpose

Our approach of using improvisational storytelling can help gather rich data on the meaning and purpose of older adults through a creative activity that is both enjoyable and socially beneficial. Personalized systems have become a focus in recent years in order to cater to individuals' unique needs and preferences. However, this information is often dispersed across multiple sources such as apps and web resources that a person uses. Moreover, there has been limited development of personalized systems specifically for older adults. Extracting this information through robots or other AI systems and questionnaires, surveys, and psychological tests can be challenging and time-consuming.

As an autonomous robot interacts with an individual over multiple storytelling sessions, it can gradually gain a deeper understanding of the person and build a relationship with them (Kanda et al., 2007). The information collected in this manner could be used to create more nuanced prompts that encourage the sharing of personal memories and experiences. Furthermore, it could enable the robot to make personalized recommendations that support the individual's meaning and purpose in life, such as suggestions on participation in community events, volunteering opportunities, social groups. It could suggest video calls with family members or friends, technology training, travel, health and wellness. At the same time, it could make recommendations for spiritual activities (meditation or prayer), recommendations for physical activities (stretching exercises, yoga, or walks in the park) based on the individual's fitness level and health conditions, and continue pursuing their existing hobbies and interests (gardening or painting).

For example, if the older adult mentions a certain location, the robot could use this information to tailor recommendations that will foster their sense of meaning and purpose. It could encourage them to further engage in conversations (“*It sounds like [Location] holds many happy memories for you. Would you like to see some pictures of the area to reminisce and reflect on these special moments?”*) or show them a video of the location (“*I noticed you have a strong connection to [Location]. How about we take a virtual tour?”*) and even use that information to strengthen their social connections (“*You seem to have a strong connection to [Location]. Would you like to reach out to friends or family members who have also been there to share memories and experiences?”*). It could connect this information with other activities that older adult values (“*It sounds like [Location] holds a special place in your heart and you enjoy painting. How about we use it as inspiration for your next painting?”*). These recommendations aim to help the older adult reconnect with the location and the emotions and memories associated with it, and in turn, foster their sense of purpose and meaning in life.

Once an autonomous system for this activity is developed, the robot could use its camera and record the sessions, generate a set of stories, and with the consent of older adults, share it with their social networks, such as their grandchildren, children, and friends with older adults' consent. This could inspire family and friends to learn about older adults' meaning and purpose, allow them to have meaningful conversations, and support them in a variety of ways. It could also increase older adults' involvement in broader community interactions and learn how their social connections value their creation, possibly leading to caregiver and family social bonding.

#### 6.3.3 Applications to other use contexts and suitable modalities

Given our technical constraints, our design of the WoZ-controlled robot was primarily targeted at individual older adults. However, future studies could also investigate how robot behaviors could encourage groups of people to share meaningful experiences and create a story together, similar to the way our participating community did with a human facilitator. The autonomous robot would need to recognize different users and learn about their meaning and purpose to motivate them individually or support social connections among them. It could also learn about the meaning and purpose of the group or the community as a collective.

Furthermore, our design could have therapeutic implications, where robots could connect certain themes and topics to give insight into a person's values (Henkel et al., 2019). It could also be used in addition to regular reminiscence therapy that can help individuals living with dementia find meaning in their personhood by promoting memory recall and emotional engagement. Digitization can potentially help address the limitations caused by carers being overly focused on tasks and residents feeling lonely in nursing homes (Tan et al., 2023), while also introducing new ideas and forms of interaction to help prevent social isolation. Previous research has also mentioned that a tele-operated android robot can facilitate communication similar to regular sessions of human-facilitated communication (Kase et al., 2019), thereby offering a scalable means of providing consistent social and emotional stimulation that supports therapeutic outcomes. As such, our design has the potential to be incorporated into technology solutions that are integrated with human interventions in memory care and treatment settings. Additionally, it can enhance interface and interaction designs among WoZ administrators, human facilitators, robotic tools, and research participants. These advancements could contribute to the development of robots with therapeutic capabilities, offering new opportunities for improving the overall wellbeing and quality of life for older adults in various care contexts. Nevertheless, our activity may also be tested for people who may benefit from reflecting on various sources of meaning and purpose in their life, such as those with temporary or situational physical and socioeconomic disabilities, reduced cognitive function, memory issues, and learning delays, those with adverse past experiences or psychological trauma and bereavement, and those experiencing loneliness, social exclusion, and isolation. To make it relevant for a variety of users, future research could also explore modalities other than images, such as songs, movies, and videos.

Finally, there has been much research on the use of robots for story-telling and learning for children (Ali et al., 2019). Future research can explore the potential of using an improvisational story-telling robot and experiences and aspects valuable to children of different ages to enhance their bonds with parents, caregivers, teachers, friends, and the community.

## 7 Conclusion

Our design of a Wizard-of-Oz controlled improvisational storytelling robot has shown promise in promoting reflection on activities and relationships that produce meaning and purpose among older adults. Through our evaluations, we found that older adults were engaged in the interaction with the robot and felt it was meaningful. Future work should focus on incorporating more sophisticated techniques for handling affective responses and addressing participant difficulties during storytelling sessions. The ultimate aim is to develop an autonomous robot that can encourage older adults to express their meaningful memories and imagination through storytelling, and use this data to enhance its understanding of how to promote meaning and purpose in their lives. For a glimpse into an imagined future shaped by a storytelling-enhancing robot, see our imaginary scenario illustrating what such a future could look like. So, ‘*Let's Make Up a Story*,' to see how a robot can inspire older adults to craft their narratives.

*In a warm and calm senior care center, QT (cutie), an autonomous robot equipped with a high-resolution tablet, embarked on a storytelling session with Betty, a vibrant older adult who had a passion for cycling. Prompted by a picture of cyclists on the tablet, Betty eagerly shared her real-life memories and imagined adventures with QT. As their conversation seamlessly transitioned between memory and creativity, QT skillfully guided Betty to discuss the upcoming Little 500 cycling race, igniting her excitement to watch it with friends. Betty's satisfaction and enthusiasm after the session showcased the meaningful engagement facilitated by QT. By processing the conversation, QT gained valuable insights into Betty's preferences and shared this data with caregivers, enhancing personalized care. This unique combination of advanced AI and empathetic engagement opened doors to innovative approaches in elder care, fostering deep connections and a sense of purpose among fellow residents*.

## Data Availability

Due to IRB regulations and the need to protect participants, the raw data supporting the conclusions of this article cannot be made publicly available by the authors. Further inquiries should be directed to the corresponding author/s.
