# Supplementary material for: Let's make up a story: design and evaluation of an improvisational storytelling robot to foster older adults' meaning and purpose in life
Source: Front Dement. 2025 Dec 2;4:1691140. doi: 10.3389/frdem.2025.1691140 (PMC12705619; doi:10.3389/frdem.2025.1691140)
Supplement: Supplementary file 1 [file Presentation_1.pdf]

# APPENDIX

## 0.1 Conversation flow which the wizard can choose from

Table 1 that demonstrates the conversation flow of the wizard's choices.

**Table 1.** Interaction Flow Between Robot and Participant

| Phase | Wizard choices |
|---|---|
| **Familiarization with Greetings and Small Talk** | • Greet participant (e.g., "Hello!")<br>• Ask for participant's name<br>• Small talk about their day ("How are you today?")<br>• Responses according to what participants said: "Good", "So-so", "Bad"<br>• Begin storytelling: choose between "Bingo" or "Forest" |
| **Active Listening and Prompting** | • Engage in image-related questions (e.g., "What is happening here?", "What time do you think it is?")<br>• Creative questions (e.g., "What are they doing?", "What might happen next?")<br>• Personal questions (e.g., "Have you experienced something like this?")<br>• Provide supportive prompts:<br>  • "Tell me more about it."<br>  • "How was it like?"<br>  • "That's nice!" (Compliment)<br>• Handle issues:<br>  • Technical issues → "Repeat"<br>  • Non-compliance → "There are no wrong answers" |
| **Prompt Wizard to Summarize the Story** | • Robot signals the wizard to summarize the conversation. |
| **Closure with a Story Title** | • Robot asks for a story title.<br>• Confirms and acknowledges participant's choice. |
| **Appreciation and Attention** | • Express appreciation (e.g., "Thanks for your story!")<br>• Positive feedback and attentiveness. |
| **Bid Farewell** | • Conclude the session politely (e.g., "Good-bye!") |

## 0.2 Imaginary story

The senior care center was awash with a warm, golden glow as the sun dipped below the horizon. Most of the care staff had gone home, leaving only a few with the residents who had finished their dinner and were

meandering around the center - unwinding. Standing among them was QT, an autonomous robot assigned to the center, equipped with a high-resolution tablet.

QT noticed one of the older adults Betty, a vibrant woman in her late seventies with a contagious smile and eyes that sparkled with mischief. QT's data bank remembered from previous sessions that Betty loved cycling with her friends every weekend around town. QT purposefully selected an image of the cyclists to evoke fond memories and facilitate the improvisational storytelling activity for the evening.

"Good evening, Betty," QT greeted, its voice soft and warm. "Would you like to join me for a storytelling session?"

Intrigued by the photo on QT's screen, Betty agreed. As the storytelling began, QT used nuanced prompts to encourage Betty to share her real-life memories of cycling with her friends and other times to imagine an adventure up the hill in the photo. The conversation flowed seamlessly, transitioning between reality and fiction.

As Betty spun her tales of past adventures and imaginary races up the hill, QT skillfully steered the conversation towards the upcoming Little 500 cycling race, suggesting Betty could watch it with her friends. A twinkle appeared in her eyes at the idea. By the end of the session, they had woven a captivating story, titled "Cycling Up The Memory Lane".

Betty looked satisfied, her face glowing with happiness. She thanked QT for the meaningful engagement.

"How did you find our session today, Betty?" QT asked, gathering informal feedback and prompting her to reflect on the meaning and purpose the conversation brought to her life. She responded with enthusiasm, expressing her delight at revisiting fond memories and excitement at the prospect of watching the upcoming race with her friends.

Soon, Betty went off to find her friends, excitedly inviting them for a session with QT, telling them about her enjoyable experience.

After the storytelling session, QT processed the conversation, gaining insights into Betty's likes, dislikes, and life purpose. It then shared this valuable data with the caregivers, allowing them to better tailor their care towards Betty's preferences, and also stored it for future interactions. QT's unique combination of advanced AI and empathetic engagement brought an innovative approach to elder care, ensuring the residents felt understood, appreciated, and deeply connected with their past, present, and future.
